# Factors Contributing to Adolescents’ and Young Adults’ Participation in Web-Based Challenges: Survey Study

**DOI:** 10.2196/24988

**Published:** 2021-02-17

**Authors:** Amro Khasawneh, Kapil Chalil Madathil, Heidi Zinzow, Patrick Rosopa, Gitanjali Natarajan, Krishnashree Achuthan, Meera Narasimhan

**Affiliations:** 1 Department of Anesthesiology and Critical Care Johns Hopkins University Baltimore, MD United States; 2 Departments of Industrial and Civil Engineering Clemson University Clemson, SC United States; 3 Department of Psychology Clemson University Clemson, SC United States; 4 Department of Clinical Psychology Amrita Institute of Medical Sciences Kochi India; 5 Center for Cybersecurity Systems and Networks Amrita Vishwa Vidyapeetham Amritapuri India; 6 Department of Neuropsychiatry and Behavioral Sciences University of South Carolina Columbia, SC United States

**Keywords:** web-based challenges, self-injurious behavior, behavior, integrated behavioral model, social media, challenge, adolescent, young adult, participation, survey

## Abstract

**Background:**

Web-based challenges, phenomena that are familiar to adolescents and young adults who spend large amounts of time on social media, range from minimally harmful behaviors intended to support philanthropic endeavors to significantly harmful behaviors that may culminate in injury or death.

**Objective:**

This study aims to investigate the beliefs that lead adolescents and young adults to participate in these activities by analyzing the amyotrophic lateral sclerosis (ALS) ice bucket challenge, representing nonharmful behaviors associated with web-based challenges, and the cinnamon challenge, representing web-based challenges that lead to harmful behaviors.

**Methods:**

A retrospective quantitative study was conducted with a total of 471 participants aged between 13 and 35 years who either had participated in the ALS ice bucket challenge or the cinnamon challenge, or had never participated in any web-based challenge. Binomial logistic regression models were used to classify those who participated in the ALS ice bucket challenge or cinnamon challenge versus those who did not engage in either challenge using the integrated behavioral model’s beliefs as predictors.

**Results:**

The findings showed that participants of both the cinnamon challenge and the ALS ice bucket challenge had significantly greater expectations from the public to participate in the challenge they completed in comparison with individuals who never participated in any challenge (*P*=.01 for the cinnamon challenge and *P*=.003 for the ALS ice bucket challenge). Cinnamon challenge participants had greater value for the outcomes of the challenge (*P*<.001) and perceived positive public opinion about the challenge (*P*<.001), in comparison with individuals who never participated in any challenge. In contrast, ALS ice bucket challenge participants had significantly greater positive emotional responses than individuals who never participated in any challenge (*P*<.001).

**Conclusions:**

The constructs that contribute to the spread of web-based challenges vary based on the level of self-harm involved in the challenge and its purpose. Intervention efforts could be tailored to address the beliefs associated with different types of web-based challenges.

## Introduction

### Background

More than 70% of Americans use social media platforms to post personal information, engage with posted content, and connect with others [1-4]. Adolescents and young adults were among the earliest internet and social media adopters and continue to use these websites at high levels [2,5,6]. Web-based challenges, or social media challenges, are popular phenomena, especially among adolescents and young adults, perhaps because of their frequent use of social networks. In these challenges, participants record themselves engaging in specific activities and share their experiences through social media platforms [6,7]. These challenges are ubiquitous and can be found on many social media platforms, including YouTube, Instagram, Facebook, and WhatsApp [8,9]. Although the activities involved in web-based challenges can vary from fun to fatal [10-13], they can generally be classified into 2 categories: (1) minimal harm challenges, which in some cases support a philanthropic cause such as the amyotrophic lateral sclerosis (ALS) ice bucket challenge [14], or (2) harmful challenges, which entail self-injurious behavior such as the cinnamon challenge [15]. Although the ALS ice bucket challenge has faced criticism (eg, safety concerns and waste of water), it is the most successful and influential fund-raising event to date [14]. In addition to raising more than US $115 million for ALS research [16], it is also credited for increasing public awareness about the disease [17].

In contrast, the cinnamon challenge involves swallowing a teaspoon of ground cinnamon without drinking any liquid for 60 seconds. The problem is that cinnamon does not dissolve or biodegrade in the lungs, as evidenced by animal-based laboratory studies, which experienced symptoms ranging from mild multifocal granulomatous inflammation to alveolar lipoproteinosis and alveolar cell hyperplasia [15,18-20]. The consequences are just as serious for humans because swallowing a large amount of cinnamon can cause pulmonary inflammation, allergic and irritant reactions, and even more serious situations, such as hypersensitivity-induced asthma attacks, which can be fatal [15]. However, none of these potentially fatal consequences have stopped adolescents and young adults from participating in the cinnamon challenge. As of 2013, there are more than 51,100 public YouTube clips of someone accepting this challenge, with some videos garnering more than 19 million views globally [15].

Given the significant amount of controversy concerning these web-based challenges, there is little research on the factors that lead individuals to participate in such challenges. For example, the extant literature on self-harm focuses primarily on a single challenge and its effect on public health and safety [15,21-23] or on how viewing content showing self-harm could lead to intentional self-harm by modeling the behavior of those we observe [24-26]. Furthermore, the literature on adolescent web-based risk focuses on the effects of engaging in web-based sexual and aggressive risk exposure [27,28]. To our knowledge, no quantitative research has comprehensively investigated the phenomenon of web-based challenges and why adolescents and young adults engage in these activities.

In this study, quantitative data were collected to explore adolescents’ and young adults’ exposure to web-based challenges and the determinants of their engagement with them through direct participation. The integrated behavioral model (IBM) [29] was used to investigate its generalizability to these behaviors on the web. It is important to reassess that and other existing behavioral theories concerning behaviors on the web because what may be true about traditional human behaviors may not apply to web environments [30].

### IBM as the Underlying Framework

**Figure 1:**
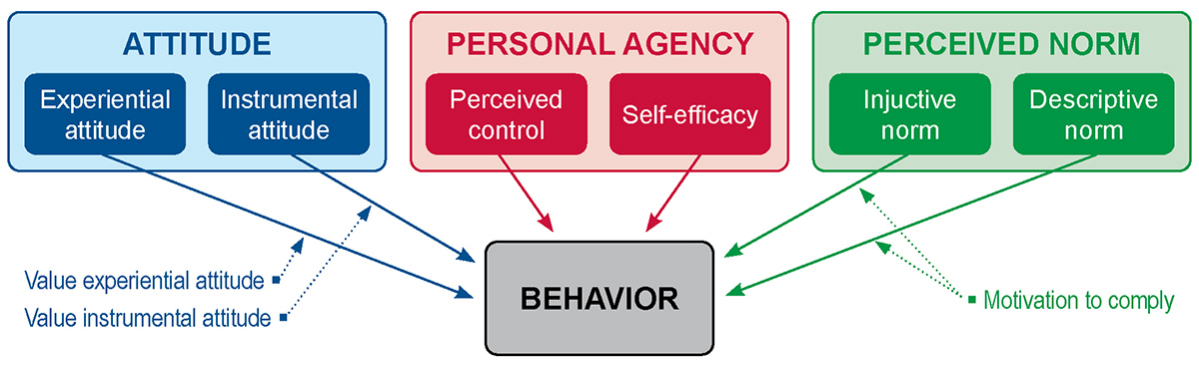
Integrated behavior model attitude.

As seen in Figure 1, IBM suggests that the intention to perform a behavior is driven by 3 factors: attitude, perceived norms, and personal agency regarding behavior. Attitude, defined as an individual preference for a certain behavioral performance, is composed of 2 dimensions: experiential attitude and instrumental attitude [31-33]. Experiential attitude is an individual’s emotional reaction to a behavior. For example, an individual with a positive emotional response toward a specific social media challenge is more likely to engage in it than an individual with a negative emotional response. Instrumental attitude is cognitively based, meaning that it is affected by a person’s beliefs about the outcomes of the behavior depending on the value of those outcomes.

Perceived norms regarding behavior and the social pressure to perform it are composed of injunctive and descriptive norms. Injunctive norms refer to the normative beliefs about others’ opinions toward participating in a challenge and the motivation to comply (if others approve or disapprove of the behavior). Descriptive norms refer to common patterns of behavior that lead to the expectations of people behaving according to that pattern.

Personal agency consists of 2 constructs: perceived control and self-efficacy. Perceived control refers to personal beliefs about the degree of control over performing the behavior. These beliefs are based on individual perceptions of how environmental factors will make the performance of the behavior difficult or easy. Self-efficacy is the individual’s certainty in their ability to perform the behavior in addition to their belief that they can overcome each prohibitive condition or obstacle [29].

### Objectives

The purpose of this study is to use IBM quantitatively to enhance our understanding of how each belief in IBM contributes to adolescents’ and young adults’ willingness to participate in web-based challenges. Another purpose of this study is to discern which beliefs are more influential than others. The findings from this study can be used to guide the development of interventions to reduce participation in harmful social media challenges among adolescent and young adult populations. Specifically, this research addressed the following research question: what is the effect, if any, of attitudes, perceived norms, and personal agency beliefs on adolescents’ and young adults’ willingness to participate in the cinnamon challenge and the ALS ice bucket challenge?

To explore our research question, we applied the IBM developed by Montano and Kasprzyk [29] depicted in Figure 1 to our hypotheses listed in Textbox 1.

**Research hypotheses.**

Research hypotheses developed based on integrated behavioral model:

- Hypothesis 1: The experiential attitude is positively related to cinnamon challenge and amyotrophic lateral sclerosis (ALS) ice bucket challenge participation.
- Hypothesis 2: The instrumental attitude is positively related to cinnamon challenge and ALS ice bucket challenge participation.
- Hypothesis 3: The value assigned to experiential attitude items moderates the relationship between experiential attitude and cinnamon challenge and ALS ice bucket challenge participation.
- Hypothesis 4: The value assigned to instrumental attitude items moderates the relationship between instrumental attitude and cinnamon challenge and ALS ice bucket challenge participation.
- Hypothesis 5: The injunctive norm is positively related to cinnamon challenge and ALS ice bucket challenge participation.
- Hypothesis 6: The descriptive norm is positively related to cinnamon challenge and ALS ice bucket challenge participation.
- Hypothesis 7: The motivation to comply moderates the relationship between the injunctive norm and cinnamon challenge and ALS ice bucket challenge participation.
- Hypothesis 8: The motivation to comply moderates the relationship between the descriptive norm and cinnamon challenge and ALS ice bucket challenge participation.
- Hypothesis 9: Perceived control is positively related to cinnamon challenge and ALS ice bucket challenge participation.
- Hypothesis 10: Self-efficacy is positively related to cinnamon challenge and ALS ice bucket challenge participation.

## Methods

### Study Overview

A survey-based study was used to investigate the application of IBM in the prediction of social media challenge behavior among adolescents and young adults. The developed survey included measures of the IBM constructs, similar to the studies reported in the literature [34-36]. The survey was pilot tested and modified accordingly. Finally, the survey was deployed to a larger sample to explore the reasons for participation in these challenges, retrospectively.

#### Measures

The dependent variable—social media participation—was collected at the beginning of the survey. The participants were asked whether they participated in the cinnamon challenge only, the ALS ice bucket challenge only, or never participated in any social media challenge. The classes for the dependent variable were balanced, with approximately one-third of the participants being in each class. The survey was then structured to include 3 main sections to assess the independent variables: a demographic section, a section related to participation in the cinnamon challenge, and a section related to participating in the ALS ice bucket challenge. The demographic section included questions about the participant’s age, gender, race or ethnicity, education, internet use, and social media challenge participation. The second and third sections assessed the following theoretical constructs related to the cinnamon challenge and the ALS ice bucket challenge: attitude, perceived norm, and personal agency. Note that the scale score for each construct was obtained by computing the mean of the relevant items.

*Attitude* was measured using 2 subconstructs: experiential attitude and instrumental attitude. Experiential attitude was measured using 4 items, each using a 7-point Likert scale. Instrumental attitude was measured using 2 items, each using a 7-point Likert scale. The value assigned to each item for both instrumental attitude and experiential attitude was measured using a 7-point bipolar scale.

*Perceived norm* was measured using 2 subconstructs: injunctive norm and descriptive norm. Each was measured using 7 items on a 7-point Likert scale. The motivation to comply construct assessed the participants’ willingness to comply with other individuals and their beliefs. This construct was measured using 7 items, each using a 7-point bipolar scale.

*Personal agency* was assessed using 2 subconstructs: perceived control and self-efficacy. Perceived control was assessed using 6 items measured on a 7-point Likert scale, whereas self-efficacy was assessed using 4 items measured on a 7-point Likert scale.

The items for these constructs were developed using the strategy suggested by Glanz et al [37] in two stages. First, a team of researchers used the data from a previous qualitative study on this topic to develop the initial set of items that measured each of the subconstructs [38]. The survey was then pilot tested using a sample of 20 participants. The results of the pilot testing were used to delete the questions that had little to no variance [29] and to improve the clarity of the remaining questions. Internal consistency reliability was calculated for each scale using Cronbach alpha (Table 1). Examples of the specific items that comprise each construct for the cinnamon challenge and the ALS ice bucket challenge are reported in Multimedia Appendix 1.

**Table 1:** Construct reliability measured using Cronbach alpha.

Construct	Cronbach alpha for Cinnamon challenge items	Cronbach alpha for ALS^a^ ice bucket challenge items
Experiential attitude	.81	.67
Instrumental attitude	.87	.69
Value assigned to experiential attitude	.85	.67
Value assigned to instrumental attitude	.92	.91
Injunctive norm	.94	.92
Descriptive norm	.91	.88
Motivation to comply	.88	.88
Perceived control	.70	.85
Self-efficacy	.66	.78

^a^ALS: amyotrophic lateral sclerosis.

#### Participants

Qualtrics Research Suite [39] was used to deploy the surveys to the participants. Inclusion criteria for the participants were participating in either the cinnamon challenge or the ALS ice bucket challenge (not both) or no participation in any social media challenge and age within the range of 13 to 35 years at the time of the study (adolescents or young adults only). A total of 471 participants completed the study. Approximately half of the participants—234 out of 471—were aged under 18 years (adolescents), and the rest—237 out of 471—were aged between 18 and 35 years (young adults), with approximately 82.6% (389/471) being females. Approximately one-third (n=153) of the respondents had participated in the cinnamon challenge only, one-third (n=155) had participated in the ALS ice bucket challenge only, and the remaining (n=163) had not participated in any social media challenge. More information about the participants is provided in Table 2.

**Table 2:** Participants’ demographics.

Variable			Values, n (%)
**Gender**			
	Female	389 (82.6)	
	Male	78 (16.6)	
	Prefer not to answer	4 (0.8)	
**Education**			
	Some high school	171 (36.3)	
	High school or GED^a^	155 (32.9)	
	2-year college degree	26 (5.5)	
	Some college	58 (12.3)	
	4-year college degree	33 (7.0)	
	Master’s degree	21 (4.5)	
	PhD degree	2 (0.4)	
	Professional degree (eg, Juris doctor or Doctor of medicine)	5 (1.1)	
**Race**			
	White	220 (46.7)	
	African American	132 (28.0)	
	Native American	4 (0.8)	
	Asian	32 (6.8)	
	Pacific Islander	2 (0.4)	
	Hispanic or Latino	55 (11.7)	
	Other	26 (5.5)	
**Employment**			
	Full-time	86 (18.3)	
	Part-time	87 (18.5)	
	Student	236 (50.1)	
	Retired	2 (0.4)	
	Unemployed	60 (12.7)	
**Age (years)**			
	<18	234 (46.7)	
	18-35	237 (50.3)	
**Social media participation**			
	Cinnamon challenge	153 (32.5)	
	ALS^b^ ice bucket challenge	155 (32.9)	
	None	163 (34.6)	
**Internet use per day (hours)**			
	<1	16 (3.4)	
	1-2	30 (6.4)	
	2-3	63 (13.4)	
	3-4	98 (20.8)	
	>4	264 (56.1)	

^a^GED: General Educational Development.

^b^ALS: amyotrophic lateral sclerosis.

#### Procedure

First, the participants read and signed the informed consent form, read the introduction to the study, and answered questions about their demographics and social media and internet use, followed by a set of screening questions. None of the participants who met the inclusion criteria based on the screening questions were ineligible to participate in the study. The screening questions were as follows:

- “Have you participated in any online challenges?”
- “Which of the following challenges did you participate in?”
- “Of the following statements, which one matches what you did in this challenge?”

**Figure 2:**
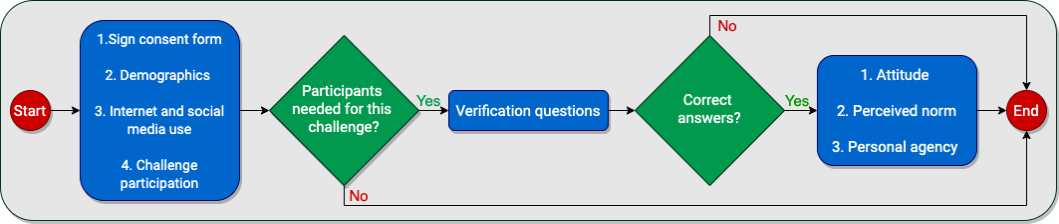
Procedure flow chart.

For the group of participants who never participated in any challenges, they had to state that in the first screening question. For the other 2 groups, if the challenge and the description did not agree, the participant was not eligible for the study. In addition, the number of participants for each challenge was restricted to having at least 75 adolescents and 75 young adults to ensure having participants from each group. The participants then answered questions to assess the constructs reported in the *Measures* section (attitude, perceived norms, and personal agency) about the cinnamon challenge and the ALS ice bucket challenge. The order of the cinnamon challenge and the ALS ice bucket challenge sections was randomly assigned to the participants. Figure 2 shows the flowchart of the study procedure. The study was approved by the Institutional Review Board of Clemson University, and all participants read and signed an informed consent form before beginning the study. Each participant was given a US $10 gift card as compensation for their time.

### Data Analysis

Binomial logistic regression was used to understand whether participation in a social media challenge (ie, either the cinnamon challenge or the ALS ice bucket challenge) can be predicted from people’s attitudes, perceived norms, and personal agency beliefs. Participation in a social media challenge is a dichotomous dependent variable (ie, 1=*participated* or 0=*did not participate*), justifying the use of the binomial logistic regression [40]. The binomial logistic regression analysis was performed using SPSS 24.0 to predict cinnamon challenge participation first with 7 predictors: age group, experiential attitude, instrumental attitude, injunctive norm, descriptive norm, perceived control, and self-efficacy. Four interaction predictors were also added to the model: experiential attitude by the value of experiential attitude, instrumental attitude by the value of instrumental attitude, the injunctive norm by the motivation to comply, and the descriptive norm by the motivation to comply. Only the interaction terms recommended by IBM were included in the analysis [29] to ensure greater power to detect significant findings within our multivariate analysis [41]. Participants who had completed the cinnamon challenge and those who did not participate in any challenge were included in this model (n=316).

A second binomial logistic regression model was used to predict ALS ice bucket challenge participation using similar predictors assessing the participants’ perception of the ALS ice bucket challenge. Participants who had completed the ALS ice bucket challenge and those who did not participate in any challenge were included in the second model. For each model, fit indices, McFadden pseudo *R^2^*, effect size estimates, estimated regression coefficients and their significance, and corresponding odds ratios and their confidence intervals were calculated.

All the data were checked to confirm the independence of observations; the existence of a linear relationship between an independent variable and the logit transformation of the dependent variable; and the absence of any multicollinearity, significant outliers, high leverage points, and highly influential points [40].

## Results

### Cinnamon Challenge

**Figure 3:**
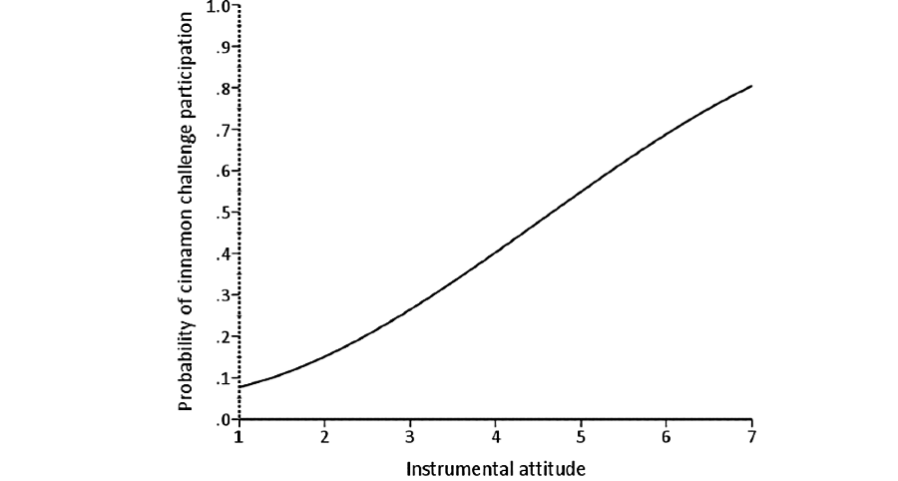
The relationship between instrumental attitude and probability of cinnamon challenge participation.

**Figure 4:**
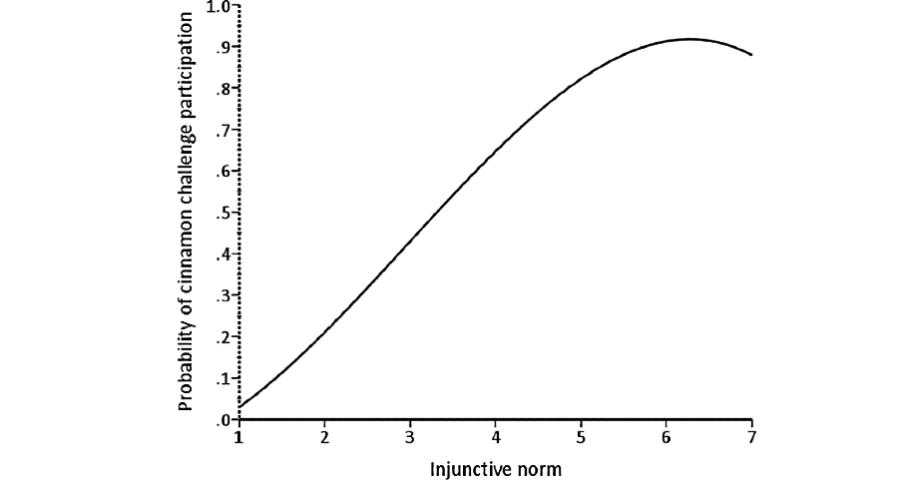
The relationship between injunctive norm and probability of cinnamon challenge participation.

**Figure 5:**
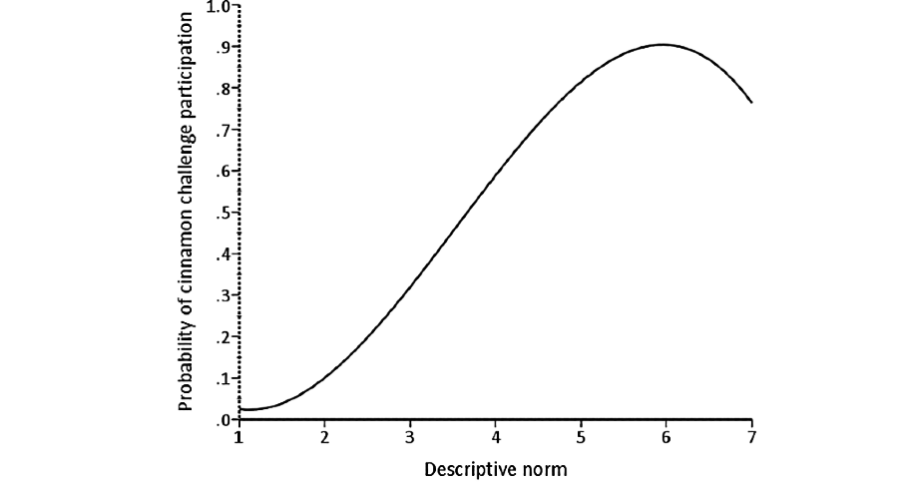
The relationship between descriptive norm and probability of cinnamon challenge participation.

The results from the direct logistic regression model predicting cinnamon challenge participation are presented in Table 3. A test of the full model with all predictors against a constant-only model was statistically significant (*χ*^2^_11_=221.8; n=316; *P*<.001), indicating that the predictors, as a set, reliably distinguished between people who had participated in the cinnamon challenge and those who had not. The deviance in participating in the cinnamon challenge accounted for by these predictors was large, with *R*^2^_L_=0.5. To test each predictor’s significance, each variable was removed from the model, and the change in *χ*^2^ was examined to determine if the removal of a variable led to a worsening of the model fit [42,43]. Independent removal of 4 of the 11 predictors significantly harmed the model fit, specifically instrumental attitude (Δ*χ*^2^_1_=11.5; *P*<.001), injunctive norm (Δ*χ*^2^_1_=30.4; *P*<.001), descriptive norm (Δ*χ*^2^_1_=6.6; *P*=.01), and the interaction term injunctive norm by the motivation to comply (Δ*χ*^2^_1_=8.8; *P*=.003). Figures 3-5 illustrate the form of these relationships.

**Figure 6:**
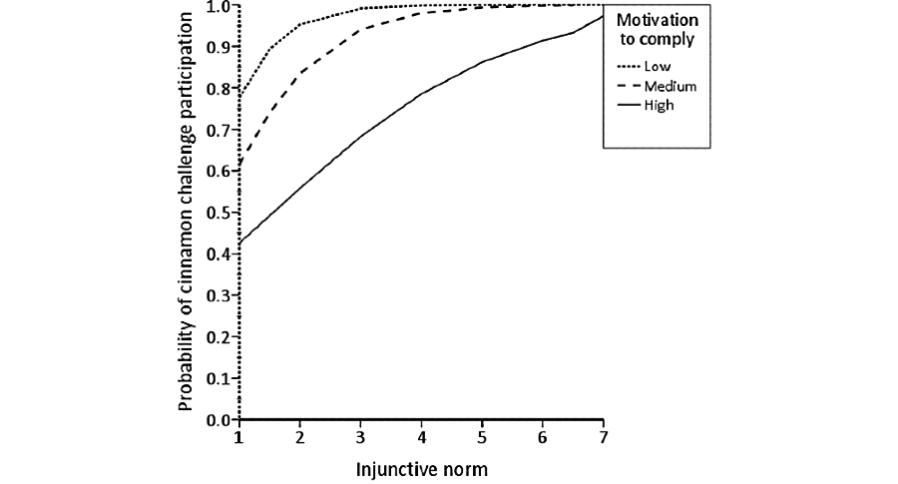
The relationship between injunctive norm and probability of cinnamon challenge participation moderated by motivation to comply.

To interpret the significant interaction for injunctive norm by motivation to comply, simple slopes were calculated from the regression coefficients at the mean of motivation to comply and 1 SD above and below the mean of motivation to comply [42]. This analysis found the slope of injunctive norm and probability to participate at the mean of motivation to comply to be *β*=1.01, and at 1 SD above and below the mean of motivation to comply to be *β*=1.80 and *β*=0.23, respectively. Figure 6 illustrates the form of this interaction.

**Table 3:** Results of binomial logistic regression model predicting cinnamon challenge participation.

Predictor^a^	*B* (SE)	Δ*χ*^2^ *(df)*	Odds ratio (95% CI)
Constant (Intercept)	−0.68 (0.29)	N/A^b^	N/A
Age group^c^	0.12 (0.35)	0.1 (1)	1.12 (0.57-2.26)
Experiential attitude	−0.31 (0.16)	4.0 (1)	0.73 (0.53-1.00)
Instrumental attitude	0.42 (0.13)	11.5 (1)**	1.52 (1.19-1.96)
Injunctive norm	1.15 (0.24)	30.4 (1)**	3.15 (2.04-5.15)
Descriptive norm	0.57 (0.23)	6.6 (1)*	1.78 (1.15-2.80)
Perceived control	0.53 (0.28)	3.9 (1)	1.71 (1.00-3.00)
Self-efficacy	0.25 (0.19)	1.7 (1)	1.30 (0.89-1.87)
Experiential attitude × value of experiential attitude	−0.09 (0.12)	0.7 (1)	0.91 (0.72-1.14)
Instrumental attitude × value of instrumental attitude	0.10 (0.07)	2.4 (1)	1.11 (0.97-1.27)
Injunctive norm × motivation to comply	−0.48 (0.16)	8.8 (1)**	0.62 (0.45-0.85)
Descriptive norm × motivation to comply	−0.12 (0.15)	0.7 (1)	0.88 (0.64-1.17)

^a^Model *χ*^2^_11_=151.05; n=318; *R*^2^_L_=0.34; null −2 Log likelihood=440.63; model 2 Log likelihood with predictors=289.58.

^b^N/A: not applicable.

^c^Age group was a dummy variable where 0=under 18 years old and 1=from 18 to 35 years old.

*P<.01; **P<.001.

### Ice Bucket Challenge

**Figure 7:**
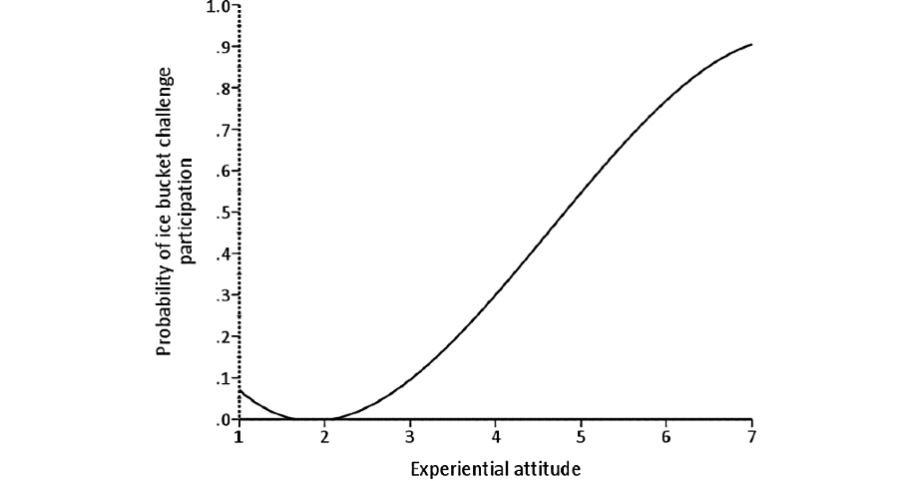
The relationship between experiential attitude and probability of amyotrophic lateral sclerosis ice bucket challenge participation.

**Figure 8:**
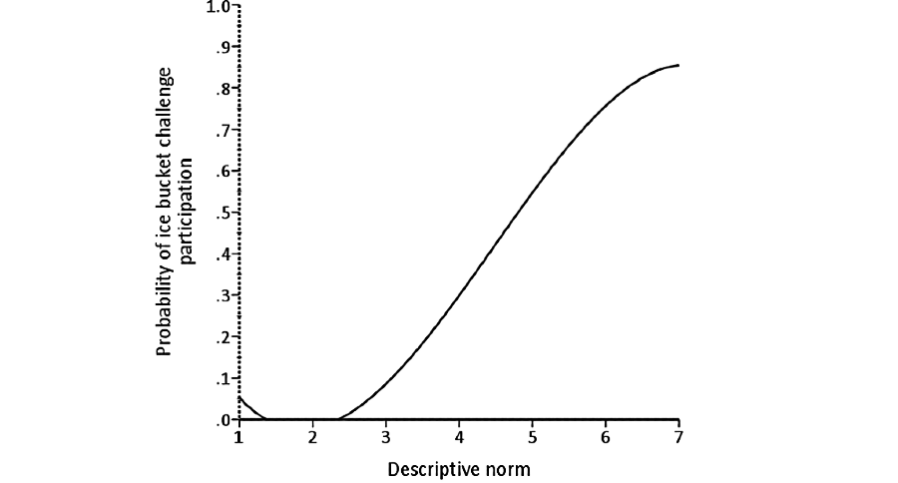
The relationship between descriptive norm and probability of amyotrophic lateral sclerosis ice bucket challenge participation.

A similar approach was used to predict ALS ice bucket challenge participation, with the results presented in Table 4. A test of the full model with all predictors against a constant-only model was statistically significant (*χ*^2^_11_=151.1; n=318; *P*<.001; *R*^2^_L_=0.34), meaning that, as a set, the predictors reliably distinguished between people who had participated in the ALS ice bucket challenge and those who had not. Independent removal of 2 of the 11 predictors significantly harmed the model fit, specifically experiential attitude (Δ*χ*^2^_1_=20.4; *P*<.001) and descriptive norms (Δ*χ*^2^_1_=9.6; *P*=.003). Figures 7 and 8 illustrate the form of these relationships.

**Table 4:** Results of binomial logistic regression model predicting amyotrophic lateral sclerosis ice bucket challenge participation.

Predictor^a^	*B* (SE)	Δ*χ*^2^ *(df)*	Odds ratio (95% CI)
Constant	0.05 (0.23)	N/A^b^	N/A
Age group^c^	−0.26 (0.30)	0.7 (1)	0.78 (0.43-1.40)
Experiential attitude	0.66 (0.16)	20.4 (1)**	1.94 (1.44-2.69)
Instrumental attitude	−0.25 (0.13)	4.0 (1)	0.78 (0.60-1.00)
Injunctive norm	0.28 (0.18)	2.4 (1)	1.33 (0.93-1.91)
Descriptive norm	0.61 (0.20)	9.6 (1)*	1.84 (1.25-2.79)
Perceived control	−0.05 (0.25)	0.1 (1)	0.95 (0.58-1.56)
Self-efficacy	0.15 (0.20)	0.6 (1)	1.16 (0.79-1.72)
Experiential attitude × value of experiential attitude	−0.15 (0.14)	1.1 (1)	0.86 (0.65-1.13)
Instrumental attitude × value of instrumental attitude	0.05 (0.07)	0.6 (1)	1.05 (0.93-1.20)
Injunctive norm × motivation to comply	0.06 (0.14)	0.2 (1)	1.06 (0.80-1.40)
Descriptive norm × motivation to comply	−0.24 (0.14)	3.0 (1)	0.79 (0.60-1.03)

^a^Model *χ*^2^_11_=151.05; n=318; *R*^2^_L_=0.34; null −2 Log likelihood=440.63; model 2 Log likelihood with predictors=289.58.

^b^N/A: not applicable.

^c^Age group was a dummy variable where 0=under 18 years old and 1=from 18 to 35 years old.

*P<.01; **P<.001.

## Discussion

### Overview

To our knowledge, this is the first study to quantitatively investigate the theoretical constructs for predicting participation in web-based challenges using data provided by actual participants. This study aimed to investigate the behavioral beliefs of people who have participated in these challenges and compare them with the beliefs of people who did not. Thus, we identified potential factors that were critical to the participants’ final decision. The results showed the attitude subconstructs, the perceived norm subconstructs, and the interaction between injunctive norm and motivation to comply to be good predictors of cinnamon challenge participation. In addition, the experiential attitude and the descriptive norm are good predictors of ALS ice bucket challenge participation.

### Cinnamon Challenge

The analysis showed that attitude and perceived norm subconstructs (hypotheses 2, 5, and 6) are strong predictors of cinnamon challenge participation. This finding is consistent with other studies that used IBM to predict other behaviors such as condom use, which also found these 2 constructs to be the strongest predictors [34,44]. As seen in the *Results* section, the relationship between instrumental attitude, injunctive norm, and descriptive norm and probability of participating in the cinnamon challenge is proportional. The positive relationship between instrumental attitude and probability of participation indicates that the more people perceive enjoyment and rewards involved in the cinnamon challenge, the more willing they were to engage in the challenge. This shows that those people thought the challenge was easy, with minimal harmful consequences. In addition, the positive relationship between injunctive norms and the probability of participation shows that the more perceived attention paid to the challenge by the public, the higher the probability of participants engaging in the cinnamon challenge because they believe their videos will receive more views. In addition, our findings suggest that there is a positive relationship between descriptive norms and the probability of participating in the cinnamon challenge. This relationship means that the less attention participants received from people around them, warning them about participating in the challenge, the higher the chance they would engage in the challenge. Consequently, it appears that the more the peers were engaging in the challenge, the higher the likelihood that participants would engage in the challenge, as they may have believed it is a common behavior that is *okay* to do. These findings are similar to a previous study on criminal behavior, highlighting the significant role that culture plays in committing crime or violent behavior [45]. In other words, in a culture where crimes occur frequently, there is a higher chance of more people committing more crimes and violent behaviors in the future.

In addition, testing hypothesis 7 showed that there is a significant interaction between injunctive norms and motivation to comply. The interaction implies that there is a positive relationship between injunctive norms and the probability of participation in the cinnamon challenge. However, this relationship is stronger for those with low motivation to comply with scores. This means that people with low motivation to comply with predominant social norms are more likely to participate in the cinnamon challenge than those with high motivation to comply. This finding is different from most of the literature on human behavior, which suggests the opposite of our findings. This is mainly because of the negative nature of the behavior that this study investigates, which involves self-harm. For example, a person with low motivation to comply with their parents is more likely to commit a self-harm behavior than someone with high motivation to comply with their parents.

Analysis of the change in model fit after removing each of the significant predictors indicated that the injunctive norm explains most of the variability in the probability of cinnamon challenge participation, followed by instrumental attitude and descriptive norm. Thus, interventions to reduce participation in similar challenges in the future should focus on these constructs, with greater emphasis on the injunctive norm, as it is the stronger predictor. This could be done by having people adolescents trust send persuasive messages highlighting the consequences of challenge behavior and explaining why they should not engage in these activities [46,47]. In addition, as there is a significant interaction between injunctive norms and motivation to comply, intervention development should consider both of these factors simultaneously. Changing only 1 of these 2 factors may lead to an undesired or unintended effect on other’s impact on challenge participation. The intervention should specifically mention the disapproval of such behaviors from those around us, even those who say they do not comply or *care* about what others say.

### Ice Bucket Challenge

Unlike cinnamon challenge participation, only the experiential attitude and descriptive norm significantly predicted ALS ice bucket challenge participation (hypotheses 2 and 6). In other words, adolescents and young adults primarily participated in this challenge for two reasons (1) enjoyment or popularity (getting more views and likes on social media) and (2) a sense of obligation due to the large number of participants completing the challenge, which made them feel that it’s the norm to do so [48-51]. We believe that other factors were not significant because of the positive nature of the challenge. For example, even people who did not participate in the challenge generally rated it as easy to perform and believed that they were capable of completing it. These beliefs may explain why perceived control and self-efficacy factors were not found to be significant predictors of ALS ice bucket challenge participation. In addition, as this challenge, in particular, was very popular, even people who chose not to participate generally indicated that everyone around them would approve of their participation. This explains why the injunctive norm factor was not found to be a significant predictor of ALS ice bucket challenge participation.

Among the significant predictors of ALS ice bucket challenge participation, experiential attitude explained the largest amount of variability, followed by the descriptive norm. These findings can help develop or market other philanthropic challenges by focusing on making them enjoyable with obvious direct rewards and emphasizing the attention given to them by the public. By developing a challenge that targets these beliefs more than the others in IBM, one could potentially create a philanthropic challenge that goes viral and leaves a health-promoting impact on society with minimal harmful consequences.

### Limitations and Future Work

This study has several limitations. Only 2 challenges were used to represent all other similar challenges. This could limit the generalizability of the findings; hence, future work could investigate the applicability of these findings to other challenges. Moreover, this study was retrospective and cross-sectional in nature, making it difficult to draw conclusions about causal relationships between the predictors and outcomes. Future work could study the impact of the constructs in controlled settings by developing interventions and examining their effects on people’s willingness to participate in social media challenges.

### Conclusions

A theoretical framework was used to guide the study design and to inform the development of theory-driven intervention efforts to change social media challenge participation intention and behavior. The cinnamon challenge was used to represent challenges with a harmful impact, and the ALS ice bucket challenge was used to represent positive-impact challenges. The constructs that were critical to the participants’ decision to participate were identified. This study provides a good theoretical model to understand the phenomenon of social media challenges. In addition, the findings provide information about which constructs should be the focus of intervention efforts. The content and thrust of those intervention efforts must be based on knowledge of how the specific items making up each construct apply specifically to social media (eg, the desire to get likes and affirmation and the social norms that are portrayed via media, videos, and images).

## Appendix

Multimedia Appendix 1 Items used to measure research constructs.

## Abbreviations

ALS: amyotrophic lateral sclerosis
IBM: integrated behavioral model

## References

[1] Lachmar E, Wittenborn A, Bogen K, McCauley H (2017). #MyDepressionLooksLike: examining public discourse about depression on Twitter. JMIR Ment Health.

[2] Demographics of social media users and adoption in the United States internet. Pew Research Center Internet & Technology.

[3] Ponathil A, Khasawneh A, Byrne K, Chalil Madathil K (2020). Factors affecting the choice of a dental care provider by older adults based on online consumer reviews. IISE Transactions on Healthcare Systems Engineering.

[4] Khasawneh A, Ponathil A, Firat Ozkan N, Chalil Madathil K (2018). How should I choose my dentist? A preliminary study investigating the effectiveness of decision aids on healthcare online review portals. Proceedings of the Human Factors and Ergonomics Society Annual Meeting.

[5] Liu S, Zhu M, Yu DJ, Rasin A, Young SD (2017). Using real-time social media technologies to monitor levels of perceived stress and emotional state in college students: a web-based questionnaire study. JMIR Ment Health.

[6] Khasawneh A, Chalil Madathil K, Dixon E, Wiśniewski P, Zinzow H, Roth R (2020). Examining the self-harm and suicide contagion effects of the Blue Whale Challenge on YouTube and Twitter: qualitative study. JMIR Ment Health.

[7] Campana MG, Delmastro F (2017). Recommender systems for online and mobile social networks: a survey. Online Social Networks and Media.

[8] Rosenthal S, Cha Y, Clark M The internet addiction test in a young adult US population. Cyberpsychol Behav Soc.

[9] Roth R, Abraham J, Zinzow H, Wisniewski P, Khasawneh A, Chalil Madathil K (2020). Evaluating news media reports on the 'Blue Whale Challenge' for adherence to Suicide Prevention Safe Messaging Guidelines. Proceedings of the ACM on Human-Computer Interaction.

[10] Mahadevaiah M, Nayak RB (2018). Blue Whale Challenge: perceptions of first responders in medical profession. Indian J Psychol Med.

[11] Burnap P, Colombo G, Amery R, Hodorog A, Scourfield J (2017). Multi-class machine classification of suicide-related communication on Twitter. Online Soc Netw Media.

[12] Karamshuk D, Shaw F, Brownlie J, Sastry N (2017). Bridging big data and qualitative methods in the social sciences: a case study of Twitter responses to high profile deaths by suicide. Online Social Networks and Media.

[13] Khasawneh A, Chalil Madathil K, Dixon E, Wisniewski P, Zinzow H, Roth R (2019). An investigation on the portrayal of Blue Whale Challenge on YouTube and Twitter. Proc Hum Factors Ergon Soc Annu Meet.

[14] Song P (2014). The Ice Bucket Challenge: the public sector should get ready to promptly promote the sustained development of a system of medical care for and research into rare diseases. Intractable Rare Dis Res.

[15] Grant-Alfieri A, Schaechter J, Lipshultz SE (2013). Ingesting and aspirating dry cinnamon by children and adolescents: the "cinnamon challenge". Pediatrics.

[16] ALS Ice Bucket Challenge Commitments. ALS Association.

[17] The ALS Association Research Program. ALS Association.

[18] Muhle H, Ernst H, Bellmann B (1997). Investigation of the durability of cellulose fibres in rat lungs. Ann Occup Hyg Internet.

[19] Tatrai E, Adamis Z, Ungvary G The pulmonary toxicity of cinnamon dust in rats. Indian J Med Res Internet.

[20] Tátrai E, Ungváry G (1992). The aetiology of experimental fibrosing alveobronchiolitis induced in rats by paprika dust. Br J Ind Med.

[21] Avery AH, Rae L, Summitt JB, Kahn SA (2016). The fire challenge: a case report and analysis of self-inflicted flame injury posted on social media. J Burn Care Res.

[22] Mukhra R, Baryah N, Krishan K, Kanchan T (2019). 'Blue Whale Challenge': a game or crime?. Sci Eng Ethics.

[23] Deklotz CMC, Krakowski AC (2013). The eraser challenge among school-age children. J Clin Aesthet Dermatol.

[24] Zhu L, Westers NJ, Horton SE, King JD, Diederich A, Stewart SM, Kennard BD (2016). Frequency of exposure to and engagement in nonsuicidal self-injury among inpatient adolescents. Arch Suicide Res.

[25] Mitchell KJ, Wells M, Priebe G, Ybarra ML (2014). Exposure to websites that encourage self-harm and suicide: prevalence rates and association with actual thoughts of self-harm and thoughts of suicide in the United States. J Adolesc.

[26] Baker DA, Algorta GP (2016). The relationship between online social networking and depression: a systematic review of quantitative studies. Cyberpsychol Behav Soc Netw.

[27] Livingstone S, Smith P (2014). Annual research review: harms experienced by child users of online and mobile technologies: the nature, prevalence and management of sexual and aggressive risks in the digital age. J Child Psychol Psychiatry.

[28] Chopik WJ (2016). The benefits of social technology use among older adults are mediated by reduced loneliness. Cyberpsychol Behav Soc Netw.

[29] Montano D, Kasprzyk D (2015). Health behavior: theory, research and practice. Public Health Behavior & Education.

[30] Gearhart S, Zhang W (2015). “Was It Something I Said?” “No, It Was Something You Posted!” A study of the spiral of silence theory in social media contexts. Cyberpsychology, Behavior, and Social Networking.

[31] Ajzen I, Fishbein M (2004). A Reasoned Action Approach: Some Issues, Questions, and Clarifications. Prediction and Change of Health Behavior: Applying the Reasoned Action Approach.

[32] French DP, Sutton S, Hennings SJ, Mitchell J, Wareham NJ, Griffin S, Hardeman W, Kinmonth AL (2005). The importance of affective beliefs and attitudes in the theory of planned behavior: predicting intention to increase physical activity1. J Appl Social Pyschol.

[33] Triandis HC (1980). Values, attitudes, and interpersonal behavior. Nebr Symp Motiv.

[34] Kasprzyk D, Montano DE, Fishbein M (1998). Application of an integrated behavioral model to predict condom use: a prospective study among high hiv risk groups1. J Appl Social Pyschol.

[35] Kasprzyk D, Montano D (2015). Application of an integrated behavioral model to understand HIV prevention behavior of high-risk men in rural Zimbabwe. Prediction and Change of Health Behavior: Applying the Reasoned Action Approach.

[36] Beville JM, Meyer MRU, Usdan SL, Turner LW, Jackson JC, Lian BE (2014). Gender differences in college leisure time physical activity: application of the theory of planned behavior and integrated behavioral model. J Am Coll Health.

[37] Glanz K, Rimer B, Viswanath K (2008). Health behavior and health education: theory, research, and practice. Health Behavior and Health Education.

[38] Khasawneh A, Chalil Madathil K, Dixon E, Wisniewski P, Zinzow H, Roth R (2019). An Investigation on the portrayal of Blue Whale Challenge on YouTube and Twitter. Proc Hum Factors Ergon Soc Annu Meet.

[39] (2015). Qualtrics XM - Experience Management Software.

[40] Hilbe J (2009). Logistic Regression Models. CRC Press.

[41] Jodoin Mg, Gierl Mj (2001). Evaluating type I error and power rates using an effect size measure with the logistic regression procedure for DIF detection. Appl Measurement Educ.

[42] Cohen J, Cohen P, West S, Aiken L (2014). Applied multiple regression/correlation analysis for the behavioral sciences. Behavioral Sciences.

[43] Moore KS, Gomer JA, Pagano CC, Moore DD (2009). Perception of robot passability with direct line of sight and teleoperation. Hum Factors.

[44] Albarracín D, Johnson BT, Fishbein M, Muellerleile PA (2001). Theories of reasoned action and planned behavior as models of condom use: a meta-analysis. Psychol Bull.

[45] Helfgott JB (2015). Criminal behavior and the copycat effect: literature review and theoretical framework for empirical investigation. Aggression and Violent Behavior.

[46] (2016). Persuasive communication. AiREAS: Sustainocracy for a Healthy City.

[47] Sparks BA, Perkins HE, Buckley R (2013). Online travel reviews as persuasive communication: the effects of content type, source, and certification logos on consumer behavior. Tourism Management.

[48] Bobo J (2007). Following the trend: Alabama abandons the duty to retreat and encourages citizens to stand their ground.

[49] Bearden WO, Etzel MJ (1982). Reference group influence on product and brand purchase decisions. J Consum Res.

[50] Cohen GL (2003). Party over policy: the dominating impact of group influence on political beliefs. J Pers Soc Psychol.

[51] Shelke S, Attar V (2019). Source detection of rumor in social network? a review. Online Social Networks and Media Internet.

