# Supplementary material for: Factors Contributing to Adolescents’ and Young Adults’ Participation in Web-Based Challenges: Survey Study
Source: JMIR Pediatr Parent. 2021 Feb 17;4(1):e24988. doi: 10.2196/24988 (PMC8078707; doi:10.2196/24988)
Supplement: Multimedia Appendix 1 [file pediatrics_v4i1e24988_app1.pdf]

Multimedia Appendix 1

Items used to measure research constructs

Construct	Sub-construct	Items	Scale
Attitude	Experiential attitude	Participating in the Cinnamon Challenge is enjoyable.	Strongly disagree-strongly agree; scored 1 to 7
		Participating in the Cinnamon Challenge makes you feel more confident.	Strongly disagree-strongly agree; scored 1 to 7
		Participating in the Cinnamon Challenge make you feel more pleasant.	Strongly disagree-strongly agree; scored 1 to 7
		Participating in the Cinnamon Challenge is for a good cause.	Strongly disagree-strongly agree; scored 1 to 7
	Vale appointed to experiential attitude	When you are challenged by someone, enjoyment is important to you.	Extremely important-extremely unimportant; scored 1 to 7
		When you are challenged by someone, feeling confident is important to you.	Extremely important-extremely unimportant; scored 1 to 7
		When you are challenged by someone, feeling pleasant is important to you.	Extremely important-extremely unimportant; scored 1 to 7
		Doing an online challenge for a good cause is important to you.	Extremely important-extremely unimportant; scored 1 to 7

Construct	Sub-construct	Items	Scale
	Instrumental attitude	Participating in the Cinnamon Challenge would get you more views than what you normally get on your posts on social media.	Strongly disagree-strongly agree; scored 1 to 7
		Participating the Cinnamon Challenge would get you more likes than what you normally get on your posts on social media.	Strongly disagree-strongly agree; scored 1 to 7
	Value appointed to instrumental attitude	Getting views on your social media posts is important to you.	Extremely important-extremely unimportant; scored 1 to 7
		Getting likes on your social media posts is important to you.	Extremely important-extremely unimportant; scored 1 to 7
Perceived norm	Injunctive	Most people would approve of you participating in the Cinnamon Challenge.	Strongly disagree-strongly agree; scored 1 to 7
		Your family would approve of you participating in the Cinnamon Challenge.	Strongly disagree-strongly agree; scored 1 to 7
		Your best friend would approve of you participating in the Cinnamon Challenge.	Strongly disagree-strongly agree; scored 1 to 7
		Your significant other would approve of you participating in the Cinnamon Challenge.	Strongly disagree-strongly agree; scored 1 to 7
		Your role model would approve of you participating in the Cinnamon Challenge.	Strongly disagree-strongly agree; scored 1 to 7
		Your friends on social media would approve of you participating in the Cinnamon Challenge.	Strongly disagree-strongly agree; scored 1 to 7
	Descriptive	Most people would participate in the Cinnamon Challenge.	Strongly disagree-strongly agree; scored 1 to 7

Construct	Sub-construct	Items	Scale
		Your family would participate in the Cinnamon Challenge.	Strongly disagree-strongly agree; scored 1 to 7
		Your best friend would participate in the Cinnamon Challenge.	Strongly disagree-strongly agree; scored 1 to 7
		Your significant other would participate in the Cinnamon Challenge.	Strongly disagree-strongly agree; scored 1 to 7
		Your role model would participate in the Cinnamon Challenge.	Strongly disagree-strongly agree; scored 1 to 7
		Your social media friends would participate in the Cinnamon Challenge.	Strongly disagree-strongly agree; scored 1 to 7
		Celebrities would participate in the Cinnamon Challenge.	Strongly disagree-strongly agree; scored 1 to 7
	Motivation to comply	You are willing to do what most people think is right.	Extremely likely-extremely unlikely; scored 1 to 7
		You are willing to do what your best friends think is right.	Extremely likely-extremely unlikely; scored 1 to 7
		You are willing to do what your family thinks is right.	Extremely likely-extremely unlikely; scored 1 to 7
		You are willing to do what your significant other thinks is right.<br>You are willing to do what your role model thinks is right.	Extremely likely-extremely unlikely; scored 1 to 7
		You are willing to do what your friends on social media think is right.	Extremely likely-extremely unlikely; scored 1 to 7
		You are willing to do what celebrities think is right.	Extremely likely-extremely unlikely; scored 1 to 7

Construct	Sub-construct	Items	Scale
Personal agency	Perceived control	Participating in the Cinnamon Challenge is easy.	Strongly disagree-strongly agree; scored 1 to 7
		The Cinnamon Challenge is doable.	Strongly disagree-strongly agree; scored 1 to 7
		Getting the tools and materials needed to participate in the Cinnamon Challenge is easy.	Strongly disagree-strongly agree; scored 1 to 7
		Going to a location where you can do the Cinnamon Challenge is easy.	Strongly disagree-strongly agree; scored 1 to 7
		Having the tools and materials needed for the Cinnamon Challenge is important.	Strongly disagree-strongly agree; scored 1 to 7
		Finding a location to do the Cinnamon Challenge is important.	Strongly disagree-strongly agree; scored 1 to 7
	Self-efficacy	Participating in the Cinnamon Challenge is under your control.	Strongly disagree-strongly agree; scored 1 to 7
		You would face barriers participating in the Cinnamon Challenge.	Strongly disagree-strongly agree; scored 1 to 7
		How certain are you that you can perform the Cinnamon Challenge?	Extremely certain-extremely uncertain; scored 1 to 7
		How confident are you that you have everything needed to perform the Cinnamon Challenge?	Extremely confident-extremely not confident; scored 1 to 7
Note: We replaced "Cinnamon Challenge" with "Ice Bucket Challenge" to these items to collect the perceived beliefs about ALS IBC.			
